# Label-free isolation of prostate circulating tumor cells using Vortex microfluidic technology

**DOI:** 10.1038/s41698-017-0015-0

**Published:** 2017-05-08

**Authors:** Corinne Renier, Edward Pao, James Che, Haiyan E. Liu, Clementine A. Lemaire, Melissa Matsumoto, Melanie Triboulet, Sandy Srivinas, Stefanie S. Jeffrey, Matthew Rettig, Rajan P. Kulkarni, Dino Di Carlo, Elodie Sollier-Christen

**Affiliations:** 1 Vortex Biosciences Inc., 1490 O’Brien Drive, Suite E, Menlo Park, CA 94025 USA; 2 Department of Bioengineering, University of California, 420 Westwood Plaza, 5121 Engineering V, PO Box 951600, Los Angeles, CA 90095 USA; 3 Department of Surgery, Stanford University School of Medicine, MSLS Bldg, 1201 Welch Road, Stanford, CA 94305 USA; 4 Department of Medicine, Stanford University School of Medicine, 875 Blake Wilbur Drive, Stanford, CA 94305 USA; 5 Departments of Medicine Urology, UCLA Medical Center, Los Angeles, CA 90095 USA; 6 Department of Medicine, VA Greater Los Angeles Healthcare System-West Los Angeles, Los Angeles, CA 90073 USA; 7 Jonsson Comprehensive Cancer Center, Los Angeles, CA 90095 USA; 8 California NanoSystems Institute, 570 Westwood Plaza, Building 114, Los Angeles, CA 90095 USA; 9 Division of Dermatology, UCLA Medical Center, 52-121 CHS, Los Angeles, CA 90095 USA

## Abstract

There has been increased interest in utilizing non-invasive “liquid biopsies” to identify biomarkers for cancer prognosis and monitoring, and to isolate genetic material that can predict response to targeted therapies. Circulating tumor cells (CTCs) have emerged as such a biomarker providing both genetic and phenotypic information about tumor evolution, potentially from both primary and metastatic sites. Currently, available CTC isolation approaches, including immunoaffinity and size-based filtration, have focused on high capture efficiency but with lower purity and often long and manual sample preparation, which limits the use of captured CTCs for downstream analyses. Here, we describe the use of the microfluidic Vortex Chip for size-based isolation of CTCs from 22 patients with advanced prostate cancer and, from an enumeration study on 18 of these patients, find that we can capture CTCs with high purity (from 1.74 to 37.59%) and efficiency (from 1.88 to 93.75 CTCs/7.5 mL) in less than 1 h. Interestingly, more atypical large circulating cells were identified in five age-matched healthy donors (46–77 years old; 1.25–2.50 CTCs/7.5 mL) than in five healthy donors <30 years old (21–27 years old; 0.00 CTC/7.5 mL). Using a threshold calculated from the five age-matched healthy donors (3.37 CTCs/mL), we identified CTCs in 80% of the prostate cancer patients. We also found that a fraction of the cells collected (11.5%) did not express epithelial prostate markers (cytokeratin and/or prostate-specific antigen) and that some instead expressed markers of epithelial–mesenchymal transition, i.e., vimentin and N-cadherin. We also show that the purity and DNA yield of isolated cells is amenable to targeted amplification and next-generation sequencing, without whole genome amplification, identifying unique mutations in 10 of 15 samples and 0 of 4 healthy samples.

## Prostate cancer: a “liquid biopsy” test for circulating tumor cells

A microfluidic device can rapidly and efficiently isolate circulating tumor cells from the blood of prostate cancer patients. Elodie Sollier-Christen of Vortex Biosciences, Rajan Kulkarni of David Geffen School of Medicine at UCLA, Dino Di Carlo of UCLA and colleagues tested the company’s microfluidic technology on blood samples taken from 21 men with advanced prostate cancer and 10 healthy controls. They showed that, within an hour, the Vortex Chip could isolate circulating tumor cells in 80% of the cancer patients and that many of these cells did not display the usual surface markers that other approaches require to capture prostate cancer cells. The purities and DNA yields of the isolated cells were high enough to enable targeted genome sequencing, which revealed mutations potentially involved in tumor formation. The Vortex technology could help diagnose prostate cancer and inform therapeutic decision-making for those with the disease.

## Introduction

Prostate cancer (PC) is currently the most common cancer among men in the world, and one of the leading causes of death from cancer in men of all races, with an estimated 26,120 deaths in 2016 in the United States alone (NCI SEER Stat Fact Sheets: Prostate Cancer). While there has been a marked increase in 5-year relative survival in the past 20 years, the majority of deaths associated with PC are attributed to failure of current therapies to cure metastatic disease. Additional research is still critically needed to address specific challenges, such as improving cancer screening to enable an earlier diagnostic, or developing more effective treatments, such as targeted therapies.

Circulating tumor cells (CTCs) are extremely rare malignant cells that originate from the primary tumor or metastatic sites and can be isolated from peripheral blood of patients with solid tumors. A few clinical trials have examined the relevance of CTC enumeration in PC^1–3^ and have shown that the number of CTCs is associated with progression-free and overall survival in advanced metastatic castration-resistant prostate cancer (mCRPC). While enumeration data provide prognostic and predictive information, it is the molecular characterization and functional analysis of CTCs that will offer insights into the biology of the tumor cells and lead to the development of personalized treatments. Genomic testing of CTCs from each patient can be performed once or repeatedly to identify certain therapeutic targets to guide the treatment for mCRPC patients or to monitor the prognosis and molecular evolution of the disease.

To date, however, the clinical utility of CTCs has been hampered by the difficulty to rapidly and effectively isolate pure populations of CTCs. Some of the pioneering technologies, including the CellSearch System, tend to be multi-steps, labor-intensive, and allow mainly for CTC detection and enumeration. More recently other technologies, such as the CTC-iChip,^4, 5^ GEDI,^6^ Adnagen,^7, 8^ and the EPIC platforms^9, 10^ have allowed the isolation and genomic analysis of CTCs from PC. However, most of these techniques require an extensive sample preparation that may lead to loss and damage of tumor cells, compromising the stability of nucleic acids for downstream analysis.^11^ Furthermore, methods such as the CTC-iChip relies on a negative depletion step to remove leukocytes, or on a positive selection step with markers such as EpCAM or other specific surface markers to capture the CTCs; this requires prior knowledge of the marker of interest for capture.^4^ The EPIC platform allows for rapid high-throughput imaging of all cells but again requires preexisting knowledge and manual selection of cells based on expression of specific biomarkers.^9^ Molecular marker-independent CTC enrichment methodologies are thus critically needed to rapidly isolate and define a broader spectrum of CTCs in PC.

To eliminate the bias associated with affinity capture, several groups have developed label-free methods for isolating CTCs.^12^ Generally, these technologies take advantage of the larger size of tumor cells of epithelial origin compared with red blood cells (RBCs) and white blood cells (WBCs) and perform size-selective isolation. The simplest of these techniques is density centrifugation, which utilizes a denser-than-water material such as sucrose gradient or Ficoll to separate cells by size and density by centrifugation through the material. While this is attractive due to its low cost and need for minimal equipment, the capture efficiency is often very low and it is difficult to get good purity as these methods are also utilized for isolation of peripheral blood mononuclear cells such as lymphocytes.^12^ This concept of size-based separation has been improved upon by several technologies. For example, the Clearbridge technology utilizes hydrodynamic sorting of cells by size in a specially designed microfluidic chip, termed Dean flow fractionation.^13^ The presence of curved channels generates rotational flow in the channel, resulting in two counter-rotating vortices across the channel cross-section (Dean vortices).^14^ Larger particles or cells (CTCs) occupy a single equilibrium position near the inner wall, while smaller cells such as RBCs or WBCs move toward the outer wall, which results in distinct cell streams that can be collected differentially. Limitations of this method include a collection into a large volume and the consequent need for additional centrifugation and transfer step.^13^

Another approach to size-based and label-free isolation is the use of microfilters to remove cells below a certain size cutoff. The isolation by size of epithelial tumor cells technology utilizes microfiltration through pores of calibrated size (usually 8 µm). Microfiltration involves flowing the blood through a device with filters of varying geometric design.^15^ One type is the membrane micro filter, which consists of a semipermeable membrane with a 2D array of small openings (between 6 and 11 µm but typically 8 µm). Smaller cells pass through, while larger ones are trapped. However, this setup is prone to clogging with cells and debris, which limits the efficiency of capture over time. A similar approach is the use of a size exclusion filter on a syringe to isolate CTCs.^16^ This method is simple and rapid and requires minimal infrastructure or equipment, though clogging and loss of cells can be a significant issue. A related approach is the Parsortix device, which functions by sorting cells by both deformability and size. The setup involves flowing blood through a microfluidic chip in which steps of varying size have been incorporated in order to separate cells by size. Cells smaller than the dimensions of the step can pass through, while those larger would remain trapped.^17^ Limitations of this approach are the need for preliminary preparation, such as buffy-coat enrichment, as well as downstream extra steps to harvest the cells from the trapping cassette.

We have previously described the Vortex Chip,^18^ a simple microfluidic device that relies on laminar microvortices to isolate and concentrate CTCs from blood or other body fluids^19^ based solely on their size. Here, we use the High Throughput Vortex Chip (Vortex HT),^20, 21^ which features an optimized processing speed (8 mL/min), higher overall capture efficiency, and high purity CTC enrichment. Using the Vortex HT chip and with objective classification criteria based on cytokeratin (CK), prostate-specific antigen (PSA), CD45, and 4,6-diamidino-2-phenylindole (DAPI) immunostaining, we were able to rapidly isolate CTCs in whole-blood samples from 22 patients with advanced mCRPC. Cells undergoing epithelial–mesenchymal transition (EMT) and which are potentially relevant in metastatic dissemination were further identified with vimentin and N-cadherin (VNC) staining. We also performed multiplex PCR-targeted next-generation sequencing (NGS) on a custom panel designed to cover all exons of four genes (AR, RB1, TP53, PTEN) and analyze the genetic variants in CTCs isolated from PC patients via NGS. At least 1 mutation was detected in 10 out of 15 (66.7%) patient samples, while no additional mutation was detected in 4 healthy donors.

## Results

### Device performance with prostate cell lines

**Fig. 1:**
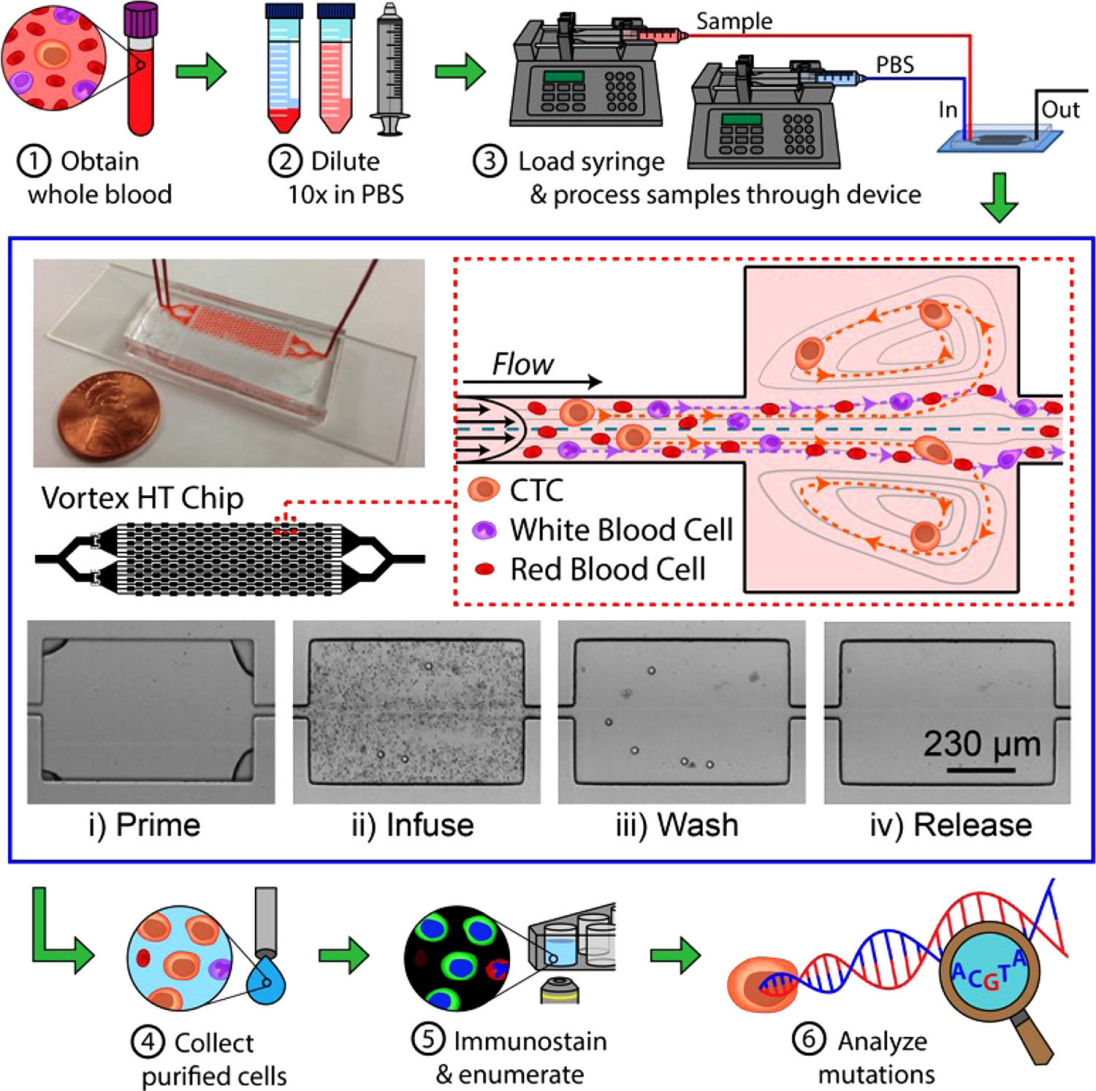
Sample processing workflow with the microfluidic Vortex HT chip. ① Whole blood samples are collected from donors in EDTA-coated tubes. ② Blood is diluted 10-fold in PBS before ③ processing through the Vortex HT microfluidic device. ④ Purified CTCs are released in a small volume and collected in a 96-well plate. ⑤ Cells are fixed, immunostained, and imaged before manual enumeration. ⑥ Mutation analysis is performed using a targeted NGS panel. The Vortex HT chip consists of 16 parallel channels and 12 serial reservoirs in each channel (*blue box*). At high flow rates (8 mL/min), laminar microvortices develop in the rectangular reservoirs and trap larger cancer cells, while smaller blood cells (RBCs and WBCs) either pass through (*red dotted box*) or are not stably trapped during the wash step. Sample processing involves (i) priming the device with wash solution to remove air bubbles, (ii) infusing the sample and capturing target cells, (iii) switching to a wash solution at the same flow rate, and (iv) releasing captured cells by lowering the flow rate to dissipate the vortices

**Fig. 2:**
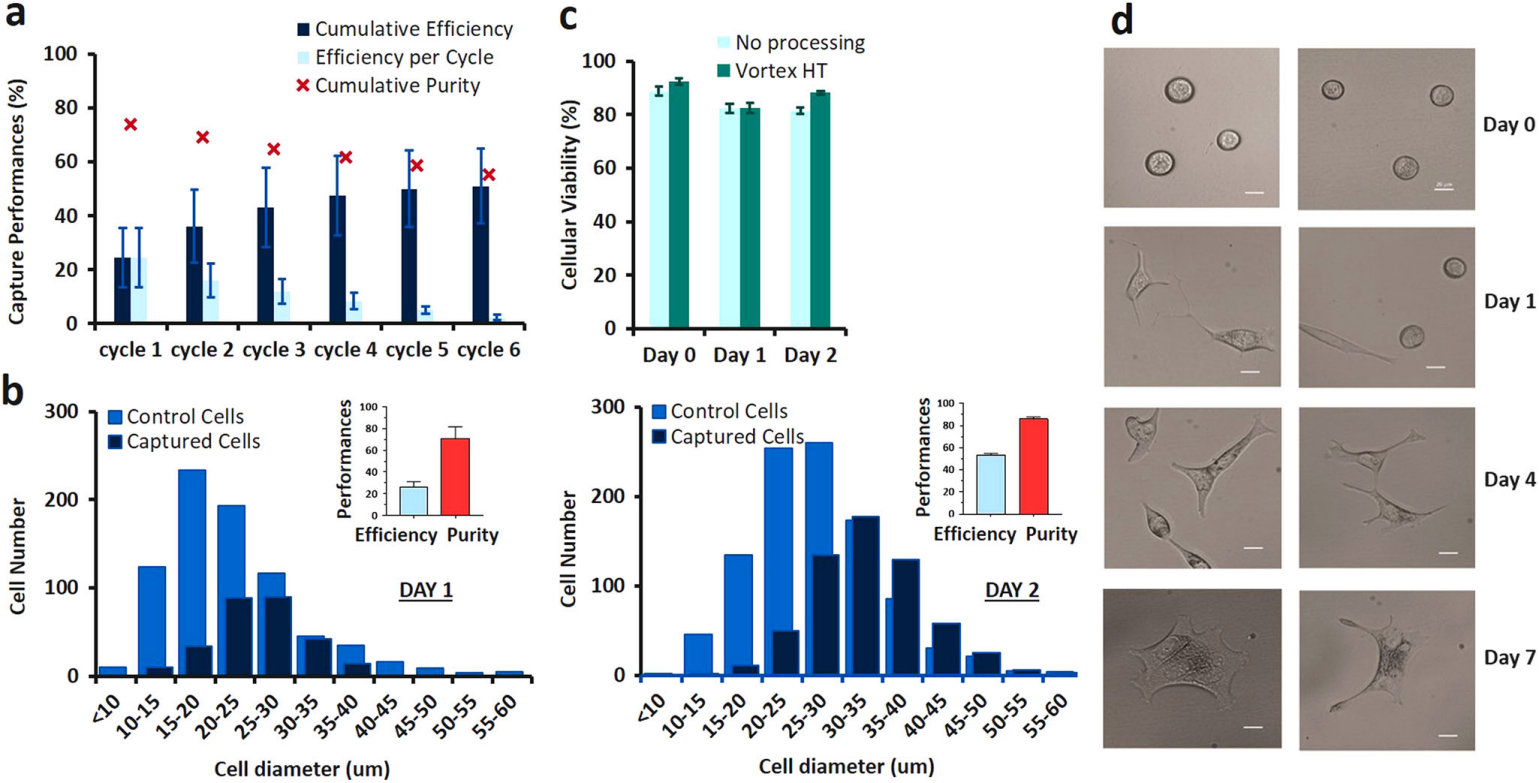
Vortex HT device performance with PC cell lines. **a** Cumulative capture efficiency and capture purity for LNCaP PC cells spiked into healthy blood. ~300 cells were spiked into 5 mL of 10X diluted blood. Experiments were performed in triplicate and repeated on 3 different days (*n* = 3). To increase cell capture, sample flow-through was collected and re-processed through the chip for additional cycles. **b** Cell diameter and capture performance at 2 different days of harvesting. **c** No difference in cell viability was observed for up to 2 days after processing through the Vortex HT chip. Unprocessed cells were used as a control. **d** After collection, LNCaP cells were maintained in culture for 7 days. Scale bar represents 20 µm

Improving upon the original design of the Vortex Chip,^18, 19^ we recently described a High Throughput Vortex Chip (Vortex HT) featuring a size cutoff of ~13 μm, with increased processing speed (8 mL/min) for the same overall capture efficiency and high purity enrichment (Fig. 1).^20^ We first evaluated the performance of the Vortex HT chip to isolate cells of PC origin (Fig. 2). Spiking experiments in phosphate buffer saline (PBS) performed with the PC cell line LNCaP resulted in a capture efficiency of 24.6 ± 7.7% on average (*n* = 12; 1 cycle) (Supp. Fig. 2). When LNCaP cells were spiked in whole blood from healthy donors, the performance remained unchanged with a 24.5% cell capture for a purity of 73.9% on average, i.e., 64.2 WBCs/mL (*n* = 3; 1 cycle) (Fig. 2a). Given the increased processing throughput (8 mL/min of 10X diluted blood) of the Vortex HT chip, in the next experiment, the sample flow-through was collected in a conical tube and reprocessed through the chip using the same operating conditions. This process was repeated for a total of six cycles and resulted in a cumulative efficiency of 51.0%, corresponding to a 2.1-fold increase when compared with a single cycle (*n* = 3). Purity of the collected cells remained high after six cycles, 55.2%, compared with 73.9% purity after one cycle (Fig. 2a). As a compromise between cell recovery and sample processing time, and to be consistent with other ongoing clinical studies, the next experiments were performed with two cycles.

To evaluate potential variability due to changes in cell properties over many culture cycles, cell lines harvested at two different time points were spiked in blood and overall cell diameter measured before and after processing through the vortex chip (two cycles total) (Fig. 2b). The overall particle diameter (i.e., from cells or cell clusters) was measured to estimate the dimension of particles entering the trapping chambers. On Day 1, cell/cell cluster diameter ranged from 8.97 to 79.03 μm, with a mean of 22.47, for a capture efficiency of 37%. On Day 2, cell/cell cluster diameter ranged from 9.39 to 66.32 μm, with a mean of 27.16 μm, for a capture efficiency of 59%. The variability observed in the distribution of the particles sizes and its impact on the resulting capture efficiencies highlights the importance of cell line maintenance and preparation^22–27^ and the limitations of using cell lines to evaluate performance of CTC capture technologies.

### Cells processed through Vortex HT chip remain viable

Live/dead assays performed over time revealed no major difference in viability between the cells processed through the Vortex HT chip and their respective controls (Fig. 2c). The cells released off-chip attached to the well-plate and were cultured for over 7 days (Fig. 2d), at which point the experiment was stopped.

### Enumeration of CTCs from PC patients and healthy subjects

CTCs were enumerated from a cohort of 20 PC samples with a median age of 71 years (range 46–87). Out of 18 patients, 17 had metastatic prostate cancer (mPC) and 1 was non-metastatic. Of the 17 mPC, 13 were mCRPC, 3 were mCSPC and the status was not available for 1 patient (Supp. Table 1). All but one patient were receiving treatment at the time of blood draw. As a control, blood from 10 healthy male donors was analyzed as well; 5 were age-matched healthy controls, with a median age of 64 years (range 46–77), and 5 were healthy volunteers under 30 years of age (median 24 years old; range 21–27) (Supp. Table 1).

**Fig. 3:**
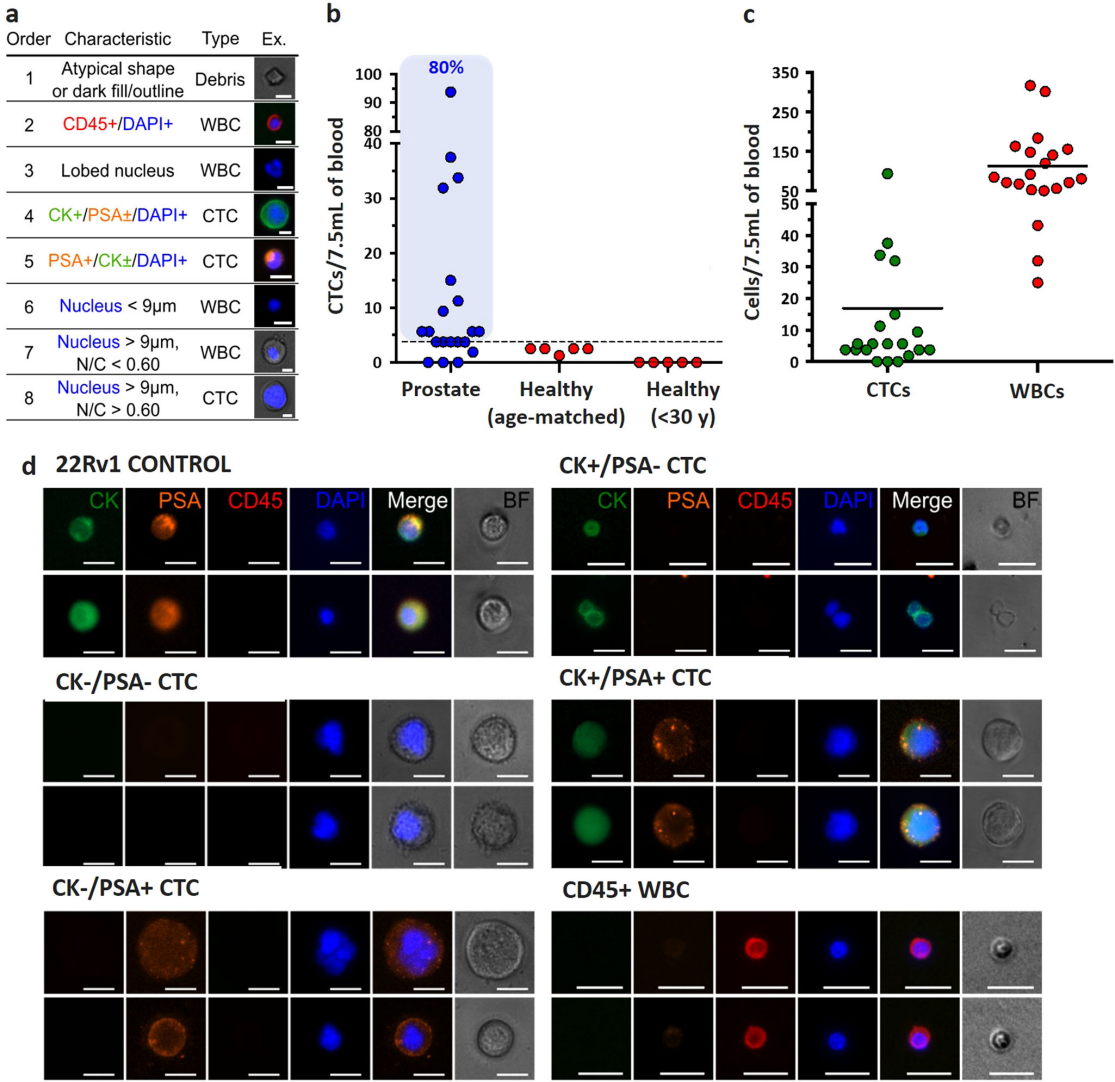
Prostate CTC enumeration criteria. **a** Collected cells were enumerated using objective criteria based on immunofluorescence staining and cytomorphological characteristics, shown in the table. Each cell was assessed for each criteria in the order listed until the characteristics matched. Scale bars represent 10 µm. **b** The number of CTCs per 7.5 mL of blood was evaluated for our PC patient cohort (*n* = 20 samples), healthy donors of same age (*n* = 5), or donors younger than 30 years old (*n* = 5). The *dotted line* represents the threshold defined from the age-matched healthy donors as the mean number of CTCs + 2 SD, calculated to be 3.37 CTCs/7.5 mL. **c** For all patient samples, CTC and WBC numbers are represented, with 0–93.75 CTCs/7.5 mL and 25.00–316.88 WBCs/7.5 mL, respectively. **d** A diverse set of CTCs were collected from patients, which varied in size and displayed all combinations of CK and PSA expression. Scale bars represent 20 µm

To identify CTCs, cells collected were immunostained with antibodies directed against CKs (CK subtypes CK1 to CK8, CK10, CK14–16, and CK19), CD45, and nuclear-stained with DAPI. The prostate origin of these circulating cells was confirmed by staining for PSA. Following imaging, cells were classified and enumerated following a set of objective criteria, and according to well-established cytomorphological features of malignancy.^20^ Detailed explanations of cell classifications with accompanying image galleries are shown in Fig. 3a and d. Briefly, CTCs were defined as nucleated (determined by DAPI staining) cells expressing either PSA or CK and lacking the marker of hematopoietic lineage CD45 (PSA+/CD45− and/or CK+/CD45−). Cells that were CD45 positive (PSA−/CK−/CD45+) were counted as WBCs. In some instances, cells were double stained for CK and CD45 (CK+/CD45+). These double-stained cells were also classified as WBCs on the basis of their multi-lobed nuclei, N/C ratio, and morphologies similar to neutrophils.

Using these enumeration criteria, 1.25–2.50 cells per 7.5 mL were isolated from the age-matched controls and characterized as CTCs (Fig. 3b). In contrast, no CTCs were found in the young healthy donor group. CTCs were detected in 17 of 20 patient blood samples (15 unique patients) with CTC counts ranging from 1.88 to 93.75 CTCs/7.5 mL (average of 16.22 CTCs/7.5 mL). Besides the CTCs, between 25.00 and 316.88 WBCs were collected per 7.5 mL (mean: 112.8 WBCs) for all patient samples considered (Fig. 3c). Using the age-matched healthy cohort enumeration data, a “healthy” cutoff value was defined as the mean number of CTCs + 2 SD, and calculated to be 3.37 CTCs/7.5 mL. Using this threshold, 16 out of 20 PC samples (80%) were considered positive for CTCs (Fig. 3b). No correlation (nonparametric Spearman correlation analysis) was found between the patients’ PSA level and CTC count (*r* = 0.1086, *n* = 20, *p* = 0.6485), or between patients’ highest Gleason score and CTC count (*r* = −0.2923, *n* = 15, *p* = 0.2869).

### CTC heterogeneity: variability in expression patterns

**Fig. 4:**
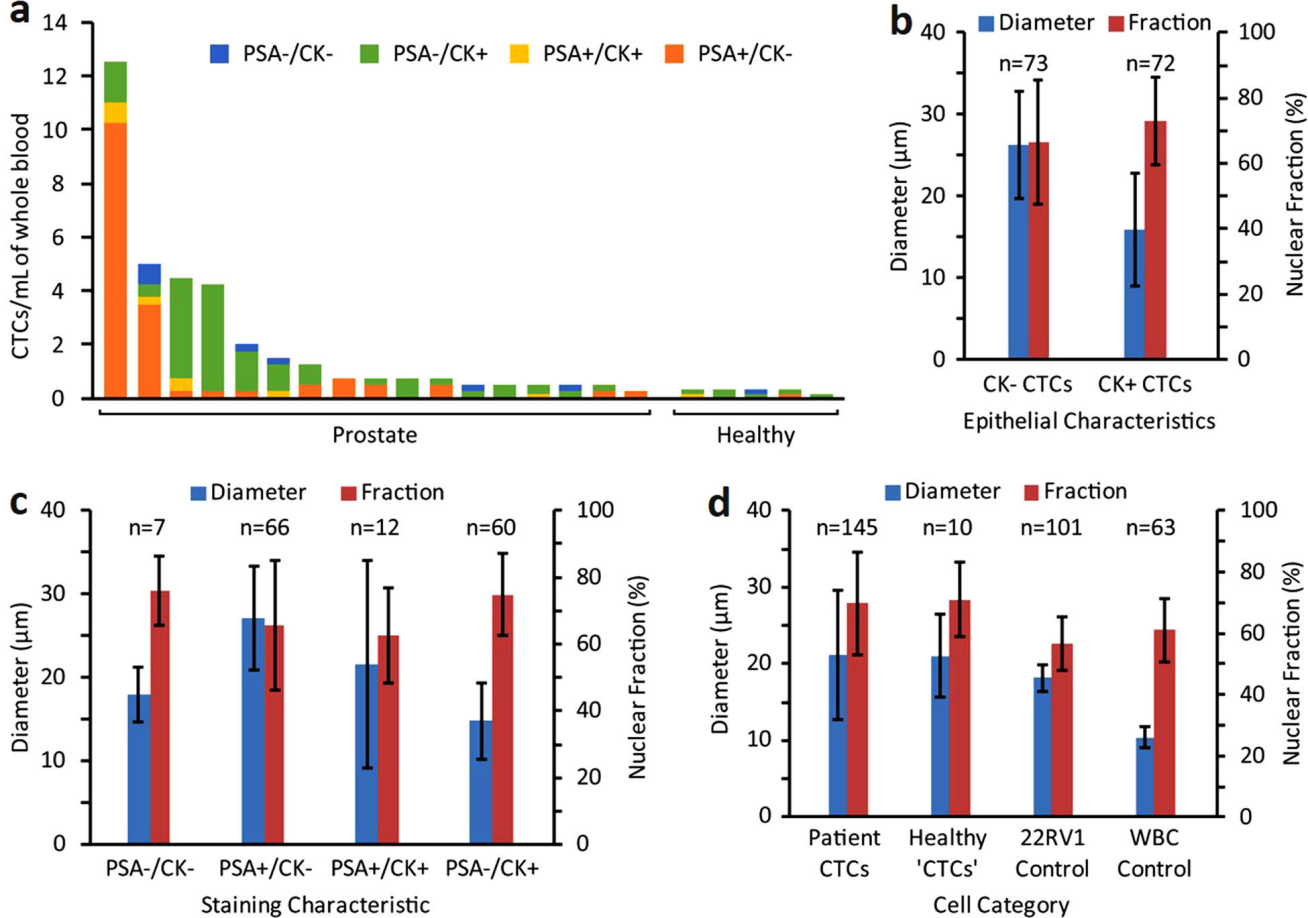
Characterization of CTC heterogeneity: expression pattern and cell morphology. **a** Collected CTCs are heterogeneous and display different combinations of PSA and CK within each patient sample. Cells classified as CTCs in healthy patient samples also exhibit all combinations of PSA and CK markers. **b** CK+ CTCs (mean 15.9 ± 6.9 µm) were significantly smaller in diameter than CK− CTCs (mean 26.2 ± 6.5 µm), which suggests potential morphological changes in cells undergoing EMT. **c** CTCs displayed all combinations of PSA and CK expression with varying size and nuclear fraction. The majority of CTCs were either PSA+/CK− (*n* = 66) or PSA−/CK+ (*n* = 60). **d** CTCs isolated from patients had similar sizes and morphologies as the few similarly classified cells found from healthy donors. CTCs displayed a wide range of sizes (mean 21.1 ± 8.4 µm), and the average diameter and nuclear fraction of CTCs were greater than those of 22RV1 PC and WBC control cells

The CTCs isolated in this patient cohort displayed varying PSA and CK expression (Fig. 4a), with 45.3% of the total CTCs collected not expressing PSA at all. Most (89.6%) of those PSA negative CTCs were expressing CK, while the remainder (10.4%) were double negative (PSA−/CK−). Regardless of PSA expression status, 51.3% of all the CTCs isolated in this study did not show CK expression. Interestingly, this trend was not observed at the individual patient level, but rather reflects a significant inter-individual phenotypic heterogeneity in our patient cohort. In healthy subjects, most of the collected cells (66.7%; 6/9 of cells collected for all healthy donors combined) were “traditional” CTCs (i.e., CK+/PSA−/CD45− cells) and 11.1% (1/9) were classified as CTCs based on cytomorphological features (CK−/PSA−/CD45− and DAPI+ only). Only two cells out of nine (22.2%) were CK±/PSA+. Approximately 48.6% of CTCs collected from patients were CK+, which is within 10% of the number found by the Vortex HT chip for non-small cell lung and breast cancer (59.3%).^20^ The lower number of CK+CTCs compared with EpCAM-based isolation approaches reflects the ability for Vortex technology to collect CTCs that have likely de-differentiated and lost epithelial characteristics.

### CTC expression of mesenchymal markers

**Fig. 5:**
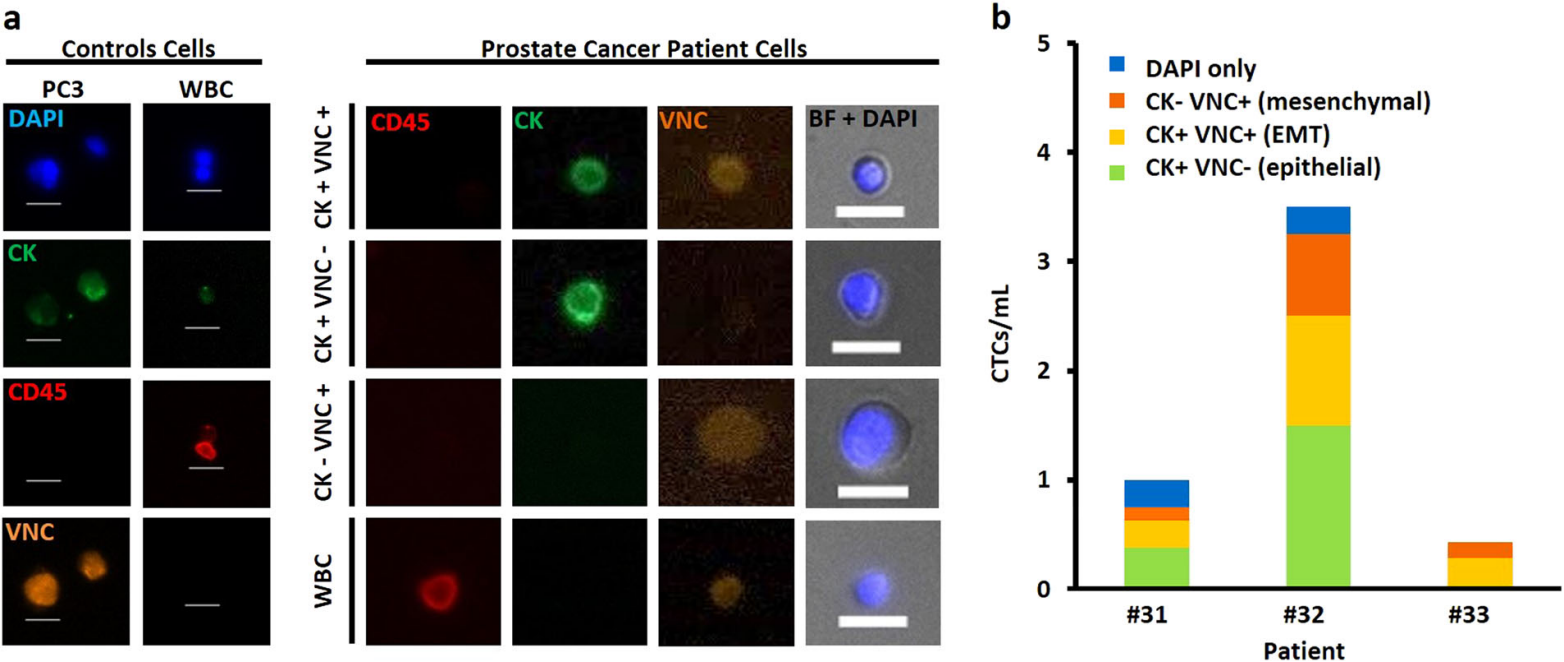
Staining with markers such as VNC identifies cells (CK negative) that may have undergone EMT. **a** Staining was optimized with PC3 prostate cell lines and WBCs as positive and negative controls, respectively, and applied to three PC patient samples. Different cell subpopulations were identified: CK+VNC+, CK+VNC−, CK−VNC+, and CK−VNC−. **b** More than 68% of the CTCs collected are CK+, more than 20% were CK−VNC+

To better characterize CTCs and gain insight into EMT in CTCs from mPCa patients, three additional blood samples were processed and the collected cells were stained with a combination of antibodies targeting VNC in addition to CK, CD45, and DAPI staining. Interestingly, prostate CTCs clustered into different subpopulations based on their CK and VNC expression pattern (Fig. 5). For patient #31, e.g., 62% of the cells collected were CK+, with 25% being both CK+ and Vim/NCad+; 12.5% additional cells were identified as mesenchymal only (CK− Vim/NCad+), and still 25% being unidentified (DAPI only, large N/C ratio). Similar percentages were obtained for patient #32, with 71% of the CTCs being CK+, 21% being only mesenchymal, and 7% not identified. Patient #33 had only CK+Vim/NCad+ and Vim/NCad+ only CTCs. A summary of the CTC characteristics is presented in Supp. Table 2.

These results show that Vortex HT can capture both cells with epithelial and mesenchymal characteristics, and thus give access to different cell phenotypes. However, the use of immunostaining only to identify the cancer cells collected has some limits, since the limited number of fluorescence filters does not allow the simultaneous staining of DAPI, CD45 for WBC exclusion, CK/Vim/NCad for epithelial and mesenchymal markers, with prostate-specific markers such as PSA. Supp. Figure 3 recapitulates the different immunostaining conditions tested, as well as the cocktail defined as optimal for our protocol and microscope set-up.

### CTC heterogeneity: morphological analysis

Cytomorphological characteristics were analyzed to explore differences between epithelial and non-epithelial CTCs, using CK staining as a marker. On average, CK− cells (26.2 ± 6.5 µm) were significantly larger than CK+ (15.9 ± 6.9 µm) cells, with no significant difference in nuclear fraction (Fig. 4b). Taking into account all combinations of CTC staining characteristics with PSA and CK, cells displayed differences in morphology but no distinct trends were observed (Fig. 4c). Since few PSA−/CK− and PSA+/CK+ cells were found, morphological differences between PSA+/CK− and PSA−/CK+CTCs were closely matched to grouped epithelial characteristics (all CK− cells vs. all CK+) discussed previously (Fig. 4b). Therefore, it is unclear whether PSA expression is correlated to cell morphology, or if morphology is more correlated to the combination of PSA and CK. Moreover, it is difficult to determine whether PSA and CK expression on CTCs were retained from cells of the original tumor or changed once cells entered circulation. To note, PSA+/CK+CTCs exhibited a uniquely wide range of cell diameters.

The sizes of CTCs matched closely between cancer patients (21.1 ± 8.4 µm) and the few identified as CTCs from healthy donors (21.1 ± 5.4 µm), as well as with 22Rv1 cancer control cells (18.2 ± 1.8 µm), and were expectedly larger than WBC (10.4 ± 1.4 µm) control cells (Fig. 4d). Patient CTCs displayed greater nuclear fractions (69.7 ± 16.7%) than 22Rv1 (56.6 ± 8.7%) and WBC (61.0 ± 10.4%) control cells, which may be due to a variety of factors. Compared with both 22Rv1 and WBC control cells, CTCs exhibited greater variance of size and nuclear fractions, which highlights the morphological differences among heterogeneous CTCs from patients. Since Vortex technology only selects for larger cells, the range of CTC sizes may be wider when taking into account smaller CTCs potentially not isolated by this approach.

### Genomics characterization of CTCs collected

Molecular characterization of cancer samples is hampered by tumor tissue availability in mCRPC patients. Access to serial liquid biopsies such as CTCs for molecular characterization would drastically change the way cancer is monitored. Here, in addition to CTC enumeration, we report the results of a mutation analysis performed on CTCs isolated from both patients and healthy donors by using a targeted NGS panel. In preliminary experiments, the workflow for multiplex PCR-targeted NGS panel was optimized and verified on LNCaP DNA (Supp. Fig. 4). First, the sensitivity of the method was verified with different amounts of DNA (1.0, 0.5, 0.2 ng, respectively) (Supp. Fig. 4C). The two mutations AR substitution c.2632 A>G (p.T878A) and PTEN deletion c.17_18delAA (p.K6fs*4) could be detected from as low as 0.2 ng input DNA (about 40 cells), with similar allelic fractions regardless of the DNA input. In a second experiment (Supp. Fig. 4D), LNCaP DNA was mixed at different ratios (80, 50, 20, 10, 5.0, 2.5, and 0%) with germline DNA (control WBC DNA) for a total of 0.5 ng DNA/sample to test the limit of detection. The AR c.2632 A>G mutation could be detected consistently in samples with as little as 5% purity. However, PTEN c.17_18delAA was detected only in samples with purity greater than 50%. This difference might reflect differential amplification in the limited DNA samples.

**Fig. 6:**
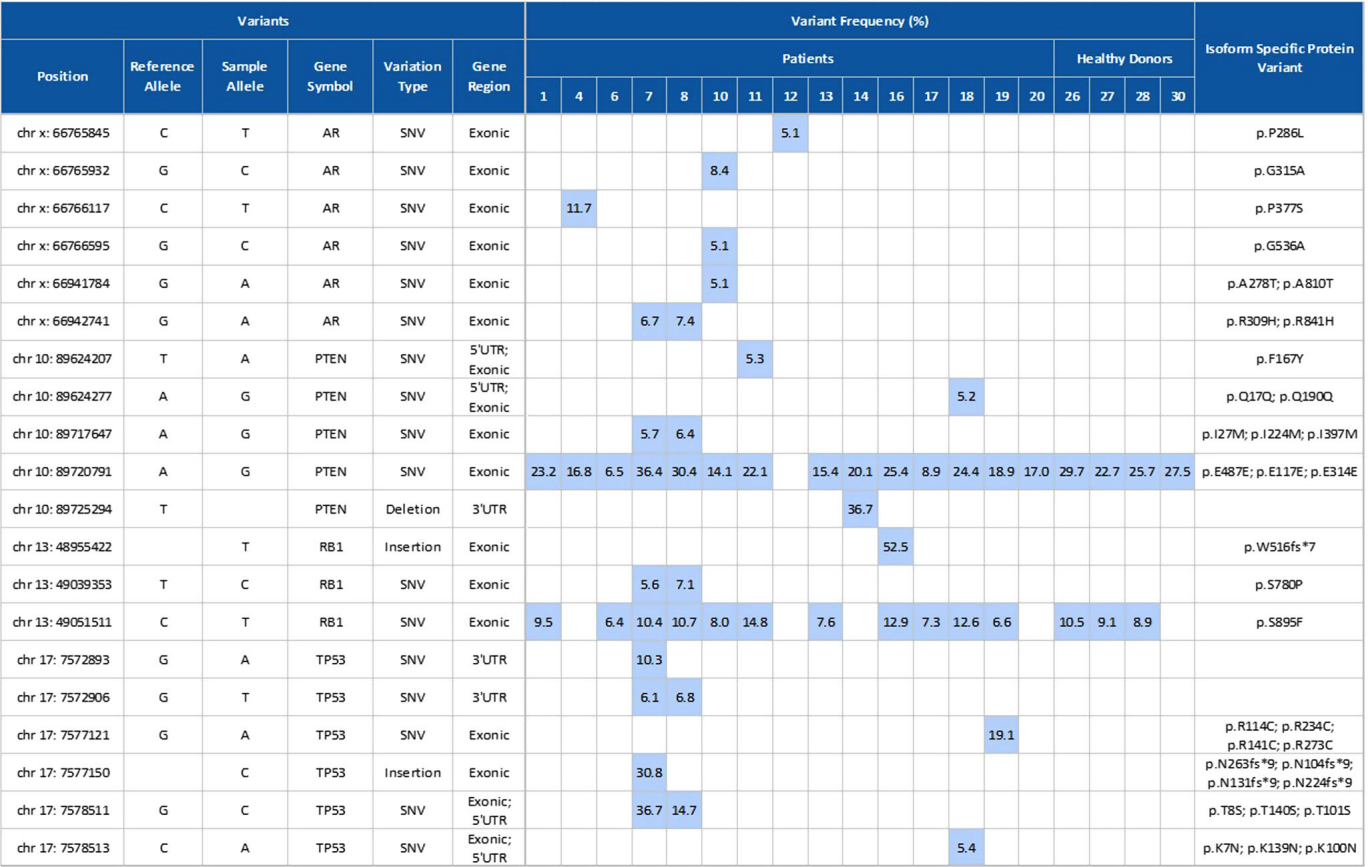
Variants and variant frequencies identified in samples isolated from PC patients and age-matched healthy donors. DNA was extracted from cells and subjected to the multiplex PCR using the GeneRead DNAseq Prostate Cancer Mix and Match Panel, enabling enrichment of the coding regions and exon/intron junctions of four genes: AR, PTEN, RB1, and TP53. Variant frequency of the detected mutation is highlighted in *light blue*

The verified workflow was then applied to cells isolated from 17 patients and 4 healthy age-matched donors. Two samples (#3 and #5) were not sequenced due to unsuccessful library preparation, leaving 15 sequenced patient samples. The remainder were sequenced, variants were called using Qiagen web portal, and further filtered by Ingenuity Variant Analysis (IVA). Finally, a total of 20 variants were detected in this cohort and are listed in Fig. 6. Among those variants, PTEN variant (chr 10: 89720791, A>G) was detected in almost all patients (14/15) and all healthy donors. RB1 variant (chr 13: 49051511, C>T) was found in 73.3% of the patients (11/15) and in 75.0% (3/4) of the healthy donors. No clear conclusion about the clinical significance of these two variants was found in the literature. Apart from these two variants, mutations were detected in 10 out of 15 (66.7%) patient samples. P53 mutation was detected in four patients, AR was detected in five patients, RB1 was detected in three patients, and PTEN in five patients. Two samples (#7 and #8), corresponding to the same patient sampled 2 months apart, showed very good concordance, with five out of seven variants being identical. No additional mutation was detected in four healthy donors.

## Discussion

In the study presented here, we describe the use of a microfluidic device for the label-free isolation of CTCs from patients with advanced PC. We find that the Vortex technology is able to isolate prostate CTCs with both high purity (from 1.74 to 37.59%, i.e., 25.00–316.88 WBCs/7.5 mL) and recovery (from 1.88 to 93.75 CTCs/7.5 mL). While some CTC technologies may require manual and time-consuming preprocessing steps, such as a RBC lysis or buffy-coat preparation,^9, 12, 13^ diluted blood can be directly processed with this microfluidic technology, hence limiting the loss of rare CTCs. Moreover, this label-free approach was able to isolate CK negative populations of CTCs in this cohort of patients and to characterize this subset of cells with additional staining for cancer-specific markers (PSA) or markers for EMT.

In this study, up to 93.75 CTCs per 7.5 mL of blood were collected, with 16.22 CTCs per 7.5 mL on average, and 112.8 WBCs, more CTCs than reported for the CellSearch system for a similar cohort of PC patients.^1^ A side-by-side comparison with CellSearch was performed by the authors with other cancer types (breast and lung) and provided a similar conclusion.^20^ Other technologies such as from Parsortix or Fluxion have reported recoveries of 33.8 and 37.6 CTCs/7.5 mL, respectively, with a median purity of 3.1 and 1% for similar patient cohort.^17^ The microfluidic-based CTC technology like iChip reported an average purity of 7.8% for positive selection (capture of EpCAM positive cells), but a contamination of around 32,000 WBCs/mL blood in the negative depletion mode (depletion of WBCs).^4^

Thus, the presented CTC platform achieves good performance, with the added advantages of a simple and fast processing time, while keeping the cells viable and intact. However, we acknowledge that there are some limitations in the current workflow. Using this microfluidic device and manual set-up, we obtained a capture efficiency of around 24.5% for LNCaP cells spiking experiments with one cycle, which highlights that some CTCs may not be effectively captured. A newer generation of this device, made of rigid plastic material, is in development to circumvent this limitation and provide higher recovery while maintaining a similar purity. Results shown here have also demonstrated that cell recovery can be improved further by multiple processing, which could be run automatically with an automated instrument. These improvements will need to be tested in a future study with more PC cell lines and patient samples. To assess the performance of our technology, a known number of cancer cell line controls was spiked into healthy donor blood. While this straightforward experimental design has been the cornerstone for the evaluation of CTC technologies so far, these results emphasize that one must be careful when using cell lines in place of CTCs as some limitations exist.^22–27^ This highlights the challenge to have a systematic and controlled method to reproducibly prepare cancer cell lines for evaluating CTC technologies without any bias. Programs such as CancerID in Europe/USA are being set-up among the community to standardize protocols. However, even though such methods were available and applied, recent studies have emphasized significant differences between cultured cell lines and patient-derived CTCs.^28, 29^ This confirms that cultured cell lines may not be an appropriate model for patient-derived CTCs.

Although 80% of the patient samples had >3.37 CTCs/7.5 mL above the threshold set from healthy donor controls, variations were observed in the healthy donors that correlated with age. Interestingly, zero CTCs were observed in the young healthy donors (<30) but 2.25 CTC/7.5 mL on average were enumerated in the age-matched (range 46–77) samples. We believe that this is the first time such a distinction has been made based on the age of the healthy donors. Besides highlighting the need for standardized controls in future CTC studies, this raises questions concerning the potential malignant origin of these cells. Although PC can be diagnosed in some very young men and at an increasing rate with age, symptom development and clinical diagnosis mostly occur in older men, if at all. A systematic review of autopsy studies showed indeed that the estimated mean prevalence of previously undiagnosed PC increased in a nonlinear fashion from 5% at age <30 years to 59% by age >79 years. The isolation of CTCs from age-matched healthy donor samples could be an indication of covert, subclinical cancer.^30, 31^ Alternatively, age-related changes in large circulating cells with epithelial signatures may be connected with other degenerative disease processes. Further studies with a larger cohort would be needed to test these hypotheses and gain more insight into the biology of these “CTCs”.

CTC capture with Vortex technology allows evaluation of both CTC morphological and molecular heterogeneity. (1) Our morphological study highlighted several key points. On the one hand, cell size matched closely between cancer patients CTCs, healthy donor “CTCs”, and control cells (22Rv1), while being all larger than WBCs as expected. On the other hand, however, cell nuclear fraction was higher for CTCs than 22Rv1 (on average 69.7 ± 16.7 vs. 56.6 ± 8.7%). Interestingly, CK negative CTCs were larger than CK positive CTCs (on average 26.2 ± 6.5 vs. 15.9 ± 6.9 µm), with no significant difference in nuclear fraction. CK negative CTCs may potentially represent cells that have undergone EMT, and an increase in average cell size agrees with observations from other studies.^32^ Larger cell size is also connected to chromosomal abnormalities and aneuploidy, which is associated with chromosomal instability, more rapid evolution, and may indicate a worse prognosis.^33^ Further long-term studies with large CTCs may shed light on correlations between average cell size and patient outcome. Differences in nuclear ratio may be due to a variety of factors, including a high degree of transcriptional activity, nuclear restructuring, cell growth, or other changes that may be associated with EMT^34, 35^ and increased invasiveness. At this stage, making additional conclusions on relationships between PSA and CK expression on CTCs and their morphologies would require a larger cohort of patients. Similarly, it would be very interesting next to apply similar morphological studies to single CTCs and clustered CTCs. (2) Immunostaining for markers such as CK, PSA, or Vim/NCad enabled the observation of multiple CTC subtypes in this patient cohort, including CK+/PSA± and CK−/PSA±. Despite CK being the standard for CTC classification, more than half (51.3%) of the CTCs isolated with the Vortex chip did not show any CK expression. This may be biased by the high CTC count of the first two patient samples. While the biological relevance of CK negative CTCs is still unclear, it has been shown that CTCs of epithelial origin can display a range of both epithelial and non-epithelial gene biomarker signatures. Indeed, in solid tumors, EMT is a process characterized by upregulation of mesenchymal markers (vimentin, fibronectin, and N-cadherin) concurrent with E-cadherin downregulation. Emerging data suggest that this phenomenon of epithelial plasticity, encompassing both reversible mesenchymal transitions and acquisition of stemness traits, may underlie the biology of tumor dissemination and treatment resistance.^36, 37^ In addition to CK expression, CTCs isolated from three mPCA patients were characterized for VNC expression. While very limited conclusions can be drawn from such a small study, it is worth noting that CTCs isolated from advanced PC patients clustered into epithelial CTCs (CK+/VNC−; 36%), EMT CTCs (CK+/VNC+; 32%), and mesenchymal CTCs (CK−/VNC+; 20%). The remaining CTCs (12%) were both CK and VNC negative. So far, CTC studies in EMT have been limited by the fact that most CTC capture technologies are dependent on epithelial marker expression (e.g., EpCAM/CellSearch). The development of label-free CTC isolation platforms, unbiased for molecular characteristics, together with the functional characterization of CTCs should definitely help elucidate the EMT process and clarify cancer metastasis mechanisms. Similarly, such technologies make possible the use of cancer-specific markers for in-depth biomarker studies.

The advantages of Vortex technology open up new opportunities. As CTC capture with Vortex technology is a label-free and contact-free process, isolated CTCs remain unbiased by molecular characteristics. CTCs are unaltered by physical filters, labels, or reagents. Experiments with cancer cell lines confirm the cells are viable, and we presume CTCs do as well, making them ideal for in vitro culture and live cell assays. This CTC isolation approach opens up possibilities to characterize the behavior and function of captured CTCs, to obtain information on proliferative and invasive properties or, ultimately, tumor re-initiating potential and response to drug. The cells are released free-floating off the microfluidic chip in a small volume and can be easily collected in a multiwell plate, microscope slide, or vial for compatibility with downstream assays (immunostaining, fluorescence in-situ hybridization, confocal, genomic assays).^21^

In addition to their use for live cell assays, CTCs provide a readily accessible source of genomic tumor material from cancer patients and may be used in lieu of tumor biopsy for cancer prognosis, disease monitoring, and targeted therapeutics. Molecular profiling of these rare cells can lead to insight on disease progression and therapeutic strategies. For example, future studies with castration-resistant prostate cancer patients will consider AR-V7 mutation as a target.^38^ However, since CTCs are rare, a significant enrichment and sample integrity is required to adapt to conventional analytical techniques. As we show, CTCs collected at a higher purity increase the accuracy and sensitivity of the downstream genomic workflow, making possible assays like Sanger sequencing,^39^ NGS with small targeted panels (present study), or larger panels. Here, a 137 amplicon panel was used on small amounts of DNA input as low as 0.2 ng, without whole-genome amplification (WGA), and still enabled the detection of mutations from CTCs collected from a blood volume as low as 4 mL in a majority of patients. Mutations were detected in 10 out of 15 (66.7%) patient samples, with P53 mutation detected in four patients (26.7%). Among them, a missense mutation of P53 in exon 8 (c.817 C>T, R273C) detected in patient 19 had been previously reported in several cancer types. For example, Nigro et al. reported a high frequency of complex TP53 mutations, including TP53 c.817 C>T, in the metastases from breast cancer patients.^40^ Several TP53 mutations, including TP53 c.817 C>T, were also reported during disease progression in acute myeloid leukemia patients^41^ or in prostatic small cell neuroendocrine carcinoma, where accumulation of p53 was observed in 56% of small cell carcinomas.^42^ Other than P53, mutations of AR, PTEN were also detected both in 33.3% (5/15) of the CTC samples. Robinson et al. recently reported that the mutation frequencies of AR, TP53, and PTEN were 62.7, 53.3, and 40.7%, respectively, in tumor biopsies from a cohort of 150 mCRPC affected individuals.^43^ The frequencies from the tumor seemed slightly higher than what we observed from the CTC samples. This may be due to the different molecular characterization between CTC and tumor biopsy, or might be biased by the small number of patients enrolled in our study (15 vs. 150 patients). Further studies comparing the CTCs with a matched tumor tissue from a larger patient cohort are needed to investigate these differences. It should be noted that here we used the pooled CTC samples to detect likely cancer mutations (even in the presence of potentially contaminating WBCs that might carry germline mutations), without access to separate WBC germline controls. To address this issue, serial filtration cascades from IVA were used to arrive at the cancer-associated somatic mutations. For example, the “predicted deleterious” filtration keeps only the variants experimentally observed to be associated with a pathogenic or likely pathogenic phenotype according to computed ACMG (American College of Medical Genetics) Guidelines classification. The filtration of “Cancer-Driven Variants” keeps only variants that are found in cancer-associated events, cancer therapeutic targets, or published cancer literature. The variants reported here are likely cancer-associated pathogenic mutations. However, to be more accurate, WBC germline controls should be included in future studies when using pooled cells.

A limitation of this study was the limited blood sample volume (4 mL) available from patients leading to a limited number of CTCs available for downstream sequencing, while the downstream fixation of the cells with paraformaldehyde also decreased the DNA yield, making it challenging to use a larger panel. NGS is still possible with larger panels but would require (i) fresh, unfixed cells, (ii) a prior WGA step, and finally (iii) access to germline DNA of the patient to obtain baseline sequence polymorphism information.

## Conclusion

We have shown that CTCs can be isolated in a label-free manner from patients with advanced PC using the microfluidic vortex technology. Cells can be isolated in less than 1 h, with high purity and good cumulative efficiency. In all, 80% of patients were observed to have cells above healthy background levels. We also found that 51% of cells collected were negative for classical epithelial markers in prostate adenocarcinoma (CK), and that some of these cells were positive for EMT markers. This suggests that the Vortex chip can efficiently isolate cells that are undergoing developmental transitions and pointing to the power of the label-free isolation method described here. Further work is underway to categorize these cells expressing EMT markers and will help add to our knowledge of PC. The clinical value of monitoring CTC counts in PC has already been confirmed in various studies and use of such label-free Vortex technology would give access to more clinical information for targeted therapy selection through a workflow for genomic analysis as described here. Future studies could also expand on the analysis of CTC morphology and viability, characterizing apoptotic CTCs, membrane intact CTCs, and CTC clusters, which may offer additional prognostic information compared with enumeration alone.^44^

## Materials and methods

### Vortex microfluidic device design

As previously introduced,^18–20^ Vortex HT is a PDMS (polydimethylsiloxane) 70-μm deep microfluidic device comprising a parallelized array of 16 straight 40-μm wide channels, leading to a series of 12 rectangular trapping reservoirs (480 × 720 μm) (Fig. 1).

### Vortex microfluidic device fabrication and operation

Conventional PDMS fabrication processes were used to assemble Vortex HT devices.^18^ A photomask was printed with the microfluidic channel layout, and utilized to fabricate a master mold from a 4-inch silicon wafer coated with negative photoresist and patterned with standard photolithographic techniques (KMPR 1050 Photoresist from MicroChem Corp.). 1:10 PDMS mix was poured on the master mold, degassed, and cured at 65 °C for 21 h. Once peeled from the master mold, cut and punched, the PDMS chip was bonded to a glass slide using oxygen plasma (800 Micro RIE, at 500 mTorr, 80 W RF, for 30 s) to enclose the channels. To operate the device, flow is driven through two inlets using two syringe pumps (Harvard Apparatus), one for the sample and one for the wash (Fig. 1). After a priming step to fill in the device with PBS at 8 mL/min for 30 s, the cell sample is infused at 7 mL/min together with wash buffer at 1 mL/min to achieve cell capture (Fig. 1). To wash the cells trapped in the vortices and remove contaminating cells, the buffer flow rate is increased to 8 mL/min, while infusion of the sample is stopped. Captured cells are then released from the vortices by stopping the flow from the wash solution.

### Cell culture and viability assay

The human prostate carcinoma epithelial cell lines 22Rv1 (ATCC® CRL2505™) and LNCaP (ATCC® CRL-1740™) were used to characterize device performance, i.e., capture efficiency and purity. Cells lines were evaluated for mycoplasma contamination by PCR and authenticated by Short Tandem Repeat profiling (CellCheck 9 Plus) by IDEXX BioResearch. 22Rv1, LNCaP, and PC-3 (ATCC® CRL-1435) cells were grown in RPMI-1640 medium (Gibco) supplemented with 10% fetal bovine serum, 1% penicillin-streptomycin, and incubated at 37 °C in a 5% CO_2_ atmosphere. Viability assays were performed with the LNCaP cell line. Briefly, cells were harvested at 30–50% confluency, 500 cells spiked into 5 mL of PBS, processed through the Vortex HT device using normal operation procedures and collected in the wells of a 96-well plate. In parallel, 500 cells were directly seeded in control wells. This process was repeated three times for each time point (1 h, 24 h, 48 h). Complete media was added to the wells and the plate was incubated at 37 °C until readout. For each time point a live/dead assay was performed by adding Calcein AM (0.5 µL of a 1 mg/mL stock) and propidium iodide (2 µL of 1 mg/mL stock) to each well and incubating for 15 min at 37 °C. The well-plate was imaged (FITC, 100 ms exposure; Tetramethylrhodamine, 500 ms exposure), live and dead cells were counted, and cell viability estimated as the number of live cells over the total number of cells expressed as a percentage (#live/#live+#dead).

### Processing of PC cell lines

Adherent cells (30–40% confluency) were dissociated with TrypLE express (Gibco), resuspended in complete media, and spiked (200–500 cells) in either 5 mL Dulbecco's PBS (DPBS) or 5 mL healthy blood diluted 10× in DPBS, prior to injection through the device. The sample was processed as indicated in the Device Operation section. The captured cells were released and collected into a 96-well plate (Greiner CELLSTAR) for imaging and enumeration. To increase capture efficiency, sample flow-through was recycled in a separate conical tube and reprocessed through the device for additional cycles. Capture performance was evaluated as follows: capture efficiency was calculated as the number of captured target cells over the total number of target cells spiked into the initial blood sample, while capture purity was calculated as the number of target cells collected over the total number of captured nucleated cells. Each experiment was performed in triplicate and repeated three separate times (*n* = 3).

### Recruitment of PC patients

Eighteen PC patients were enrolled in the UCLA Oncology Clinics using Institutional Review Board approved protocol (UCLA IRB #11-01798) and three stage IV patients were enrolled from the Stanford Cancer Center (Stanford IRB #350—Protocol #5630) (Supp. Table 1). Samples #10 and 11 correspond to distinct time points of the same patient. The age of patients ranged from 46 to 87 years old. Blood was also collected from young (<30 years of age, *n* = 5, UCLA) and age-matched (*n* = 5, Stanford) healthy volunteers, and processed in the same manner as the patient samples (Supp. Fig. 1). The healthy volunteers had no known illness or fever at the time of draw, and no history of malignant disease. To be included in this study, all donors provided written informed consent. Peripheral blood sample was collected into 10 mL EDTA-coated tubes (BD Vacutainer), transported and stored at room temperature (RT), and processed within 4 h of the draw.

### Processing of PC patient samples

Whole blood (4–8 mL) was diluted 10-fold in PBS, then processed as described in the Device Operation section. CTCs were collected into a 96-well plate (Greiner CELLSTAR) for imaging and enumeration. Sample flow-through was collected in a conical tube and reprocessed through the device for a total of two cycles.

### Cell immunostaining and enumeration

Cells collected from 20 samples (18 patients) were fixed with 2% paraformaldehyde (Electron Microscopy Sciences) for 10 min, permeabilized with 0.4% v/v Triton X-100 (Research Products International Corp) for 7 min, blocked with 5% goat serum (Invitrogen) for 30 min, and labeled for 1 h at RT with DAPI (Life Technologies), anti CD45-phycoerythrin (CD45-PE, Clone HI30, BD Biosciences), and a cocktail of primary antibodies to identify CK positive cells (Pan-CK clone AE1/AE3, eBioscience, clone CK3-6H5, Miltenyi Biotec, and CK clone CAM5.2, BD Biosciences). PSA positive cells were identified using a rabbit anti-PSA polyclonal antibody (Dako) and were visualized using AlexaFluor-647 secondary antibodies (Anti-rabbit IgG, Cell Signaling). For three patient samples, PSA staining was replaced by Vimentin (Vimentin-AF467, clone V9, Abcam) and N-cadherin (NC-AF647, clone EPR1791-4, Abcam) staining. After staining, the cells were imaged (Axio Observer Z1, Zeiss) and manually enumerated using specific classification criteria (Fig. 3a, d). For patient samples, purity is calculated as the number of CTCs that meet the criteria described in Fig. 3a (i.e., DAPI+ with CK and/or PSA positivity or nuclear size >9 μm with nuclear/cytoplasmic (N:C) ratio >0.6 and negative for CD45) divided by the total number of DAPI positive cells counted.

### Cell morphology analysis

To measure average cell/cell cluster size, cancer cells were stained with Calcein AM (1 μM) in the cell flask before trypsinization, incubated for 15 min, washed with PBS, and trypsinized following the protocol provided above. Cell/cell cluster diameter was measured with Zen software from Zeiss, with the diameter being defined as the largest dimension of the object if not spherical. Immunostained cells were also assessed for morphology metrics. Zen software was used to calculate areas of line traces around cell membranes and nuclei. Nuclear fraction was defined as the ratio of nuclear area to whole cell area observed, N:C ratio was defined as the ratio of nuclear area to cytoplasmic area (cellular area minus nuclear area), and cell diameter was back-calculated from cellular area, assuming a circular cell.

### Mutation analysis

For gene mutation analysis, a Qiagen GeneRead DNAseq-targeted panel NGS was selected, optimized, and verified first on a PC cell line (LNCaP), then applied to cells isolated from patient’s samples. Genomic DNA from cells isolated from 17 PC patients and 4 age-matched healthy donors was extracted using the QIAamp Micro Kit (Qiagen). The Qiagen optimized protocol for small tissue samples was followed with some modifications. Specifically, the 96-well plate was centrifuged after cell enumeration, PBS was carefully removed from each well, and replaced by buffer ATL and proteinase K. The plate was sealed with adhesive sealing film and incubated overnight at 60 °C. The lysate was transferred to a micro centrifuge tube, and Buffer AL and carrier RNA were added to continue the lysis step. For each patient sample, lysates from multiple wells were then combined and loaded onto the provided column and the rest of the procedure followed without further modification. DNA was quantitated by absolute quantitative qPCR (7500 Fast Real-Time PCR system, Applied Biosystems®). DNA was subjected to multiplex PCR amplification using the GeneRead DNAseq Prostate Cancer Mix and Match Panel (Qiagen), containing 137 primer sets covering all exons of the following genes: AR, PTEN, RB1, and TP53. The amplified PCR products were purified with the Agencourt AMPure XP beads (Beckman Coulter), subjected to the library preparation using Truseq library prep kit (Illumina), and then sequenced on Miseq sequencer using v2 chemistry (Seqmatic, Fremont, CA). Fastq.gz files generated by the MiSeq Reporter program (Illumina) were uploaded into QIAGEN NGS data analysis web portal. The resulting analysis-ready report (.VCF file) was uploaded directly and further analyzed through the use of QIAGEN’s Ingenuity® Variant Analysis™ software (www.qiagen.com/ingenuity) from QIAGEN (Redwood City), using the following filter cascade: Variants→Confidence→Common Variants→Predicted Deleterious→Genetic Analysis→Cancer-Driven Variants.

## Electronic supplementary material

Supplementary Table 1

Supplementary Table 2

Supplementary Figure 1

Supplementary Figure 2

Supplementary Figure 3

Supplementary Figure 4

## Electronic supplementary material

Supplementary Information accompanies the paper on the *npj Precision Oncology* website (doi:10.1038/s41698-017-0015-0).
