# Supplementary figures and images for: Label-free isolation of prostate circulating tumor cells using Vortex microfluidic technology
Source: NPJ Precis Oncol. 2017 May 8;1:15. doi: 10.1038/s41698-017-0015-0 (PMC5859469; doi:10.1038/s41698-017-0015-0)

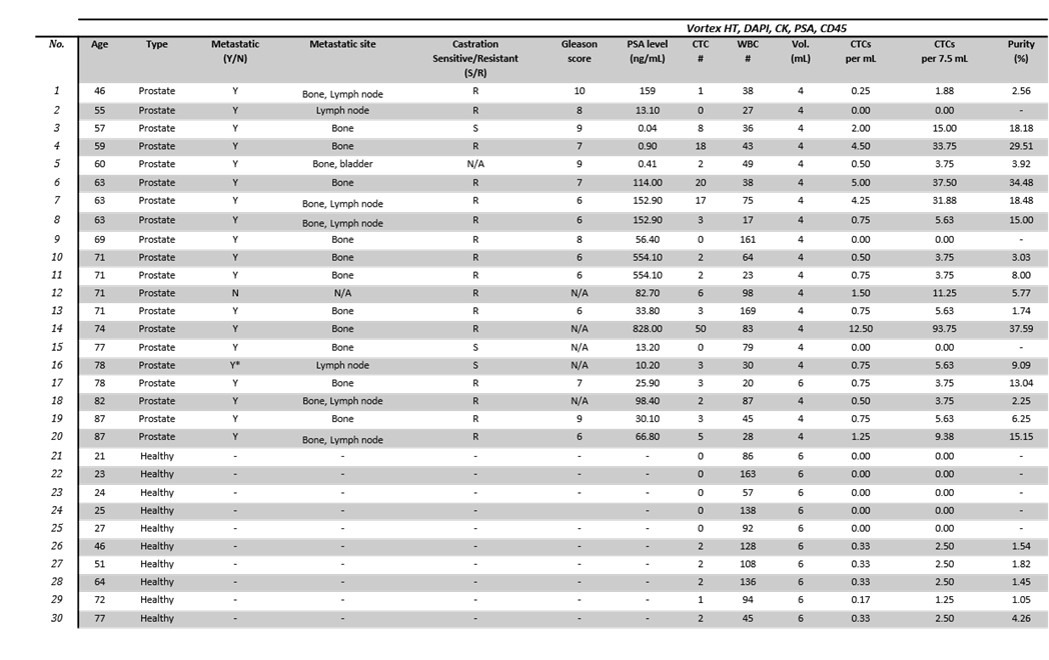

Supplement: Supplementary file 1 — Supplementary Table 1 [file 41698_2017_15_MOESM1_ESM.jpg]

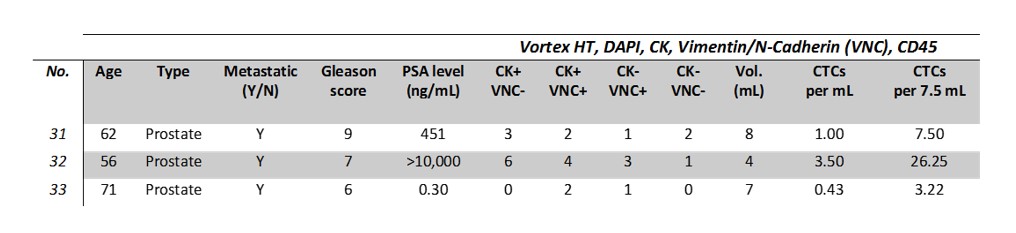

Supplement: Supplementary file 2 — Supplementary Table 2 [file 41698_2017_15_MOESM2_ESM.jpg]

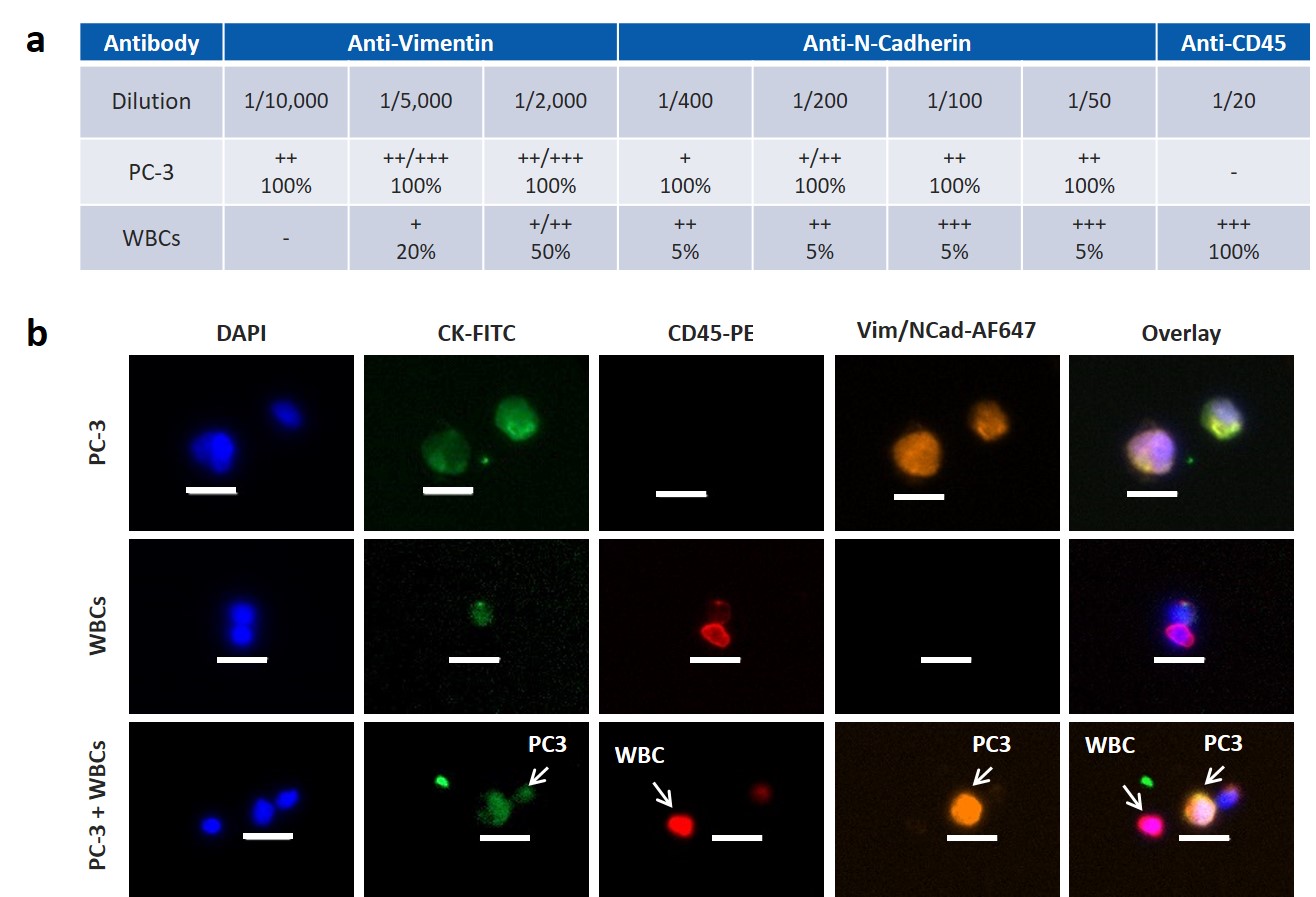

Supplement: Supplementary file 3 — Supplementary Figure 1 [file 41698_2017_15_MOESM3_ESM.jpg]

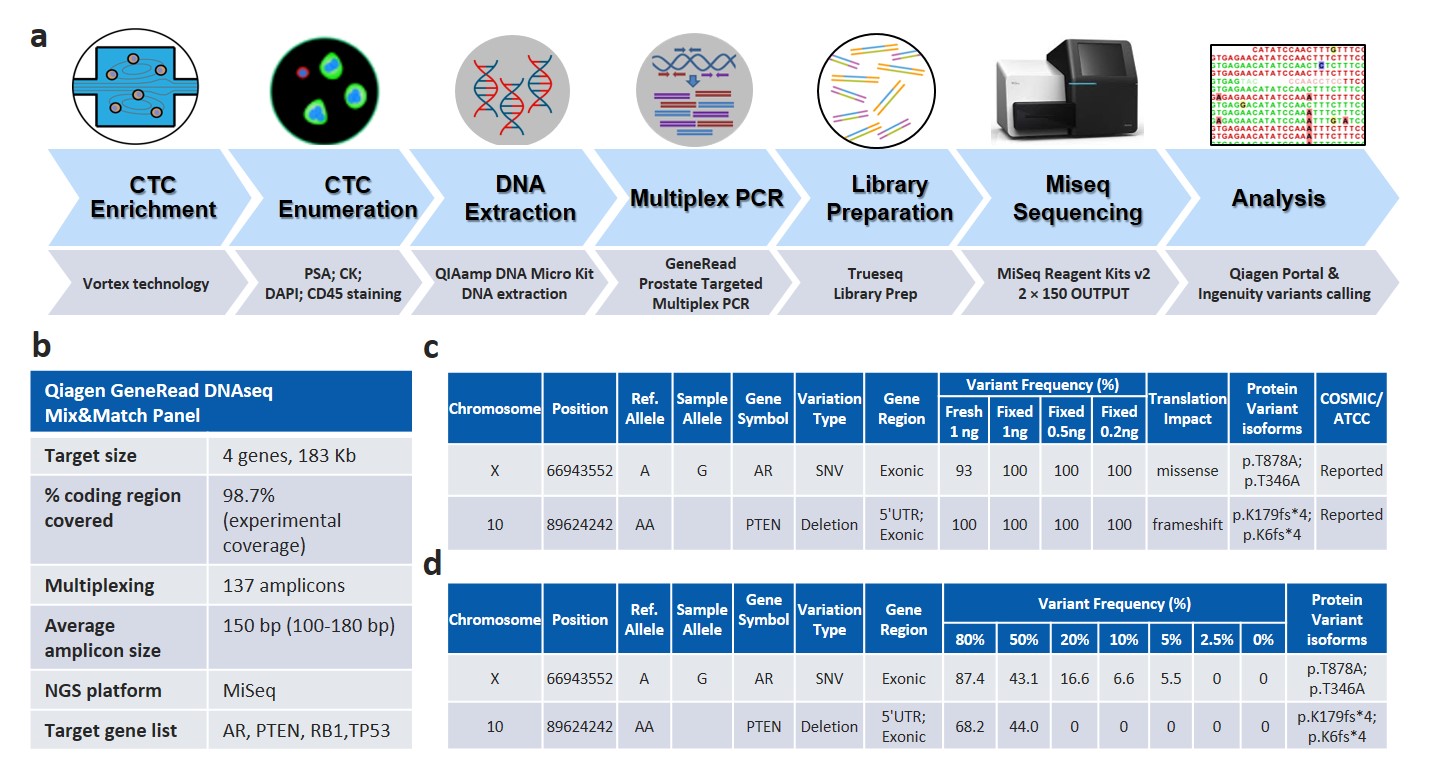

Supplement: Supplementary file 4 — Supplementary Figure 2 [file 41698_2017_15_MOESM4_ESM.jpg]

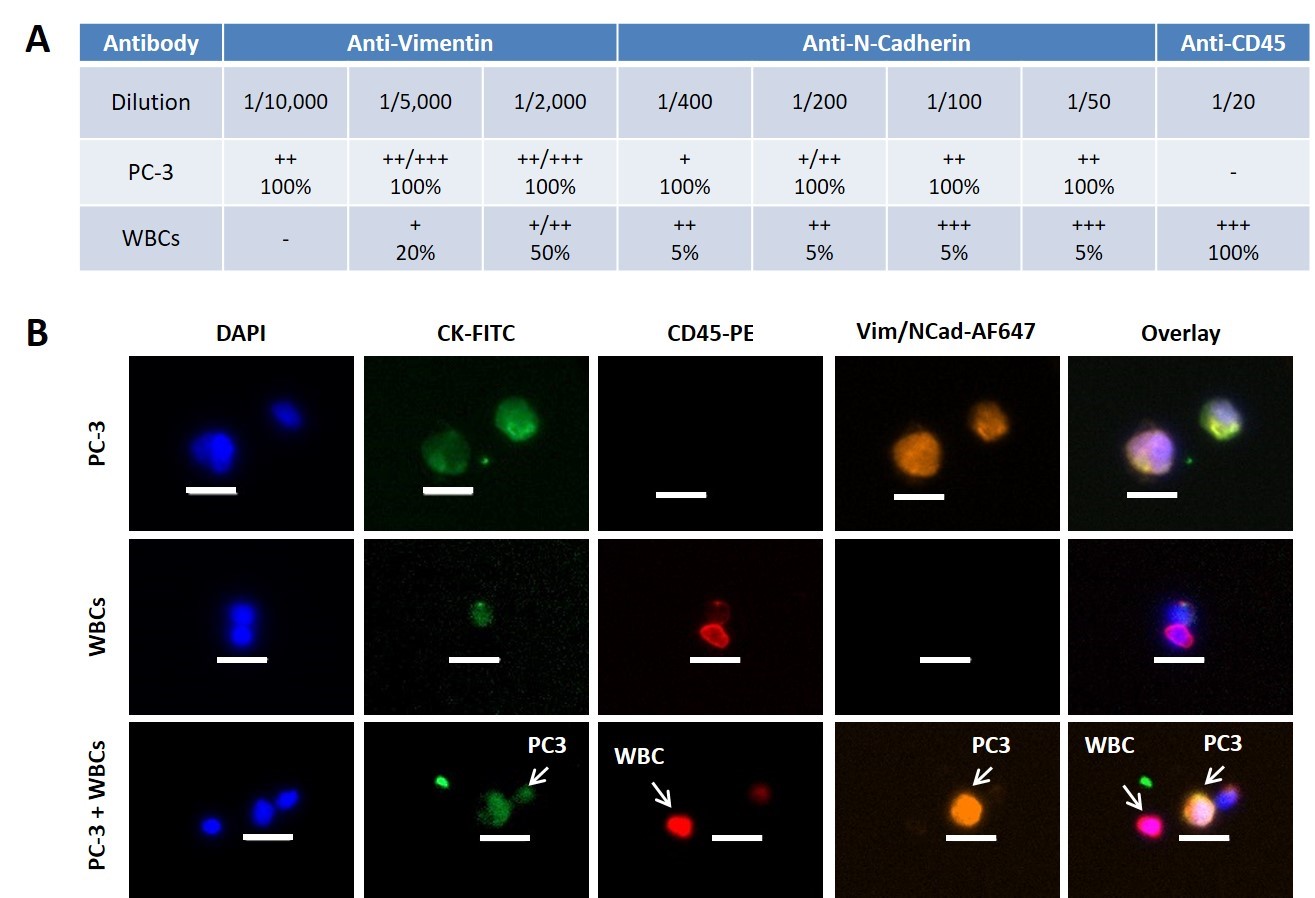

Supplement: Supplementary file 5 — Supplementary Figure 3 [file 41698_2017_15_MOESM5_ESM.jpg]

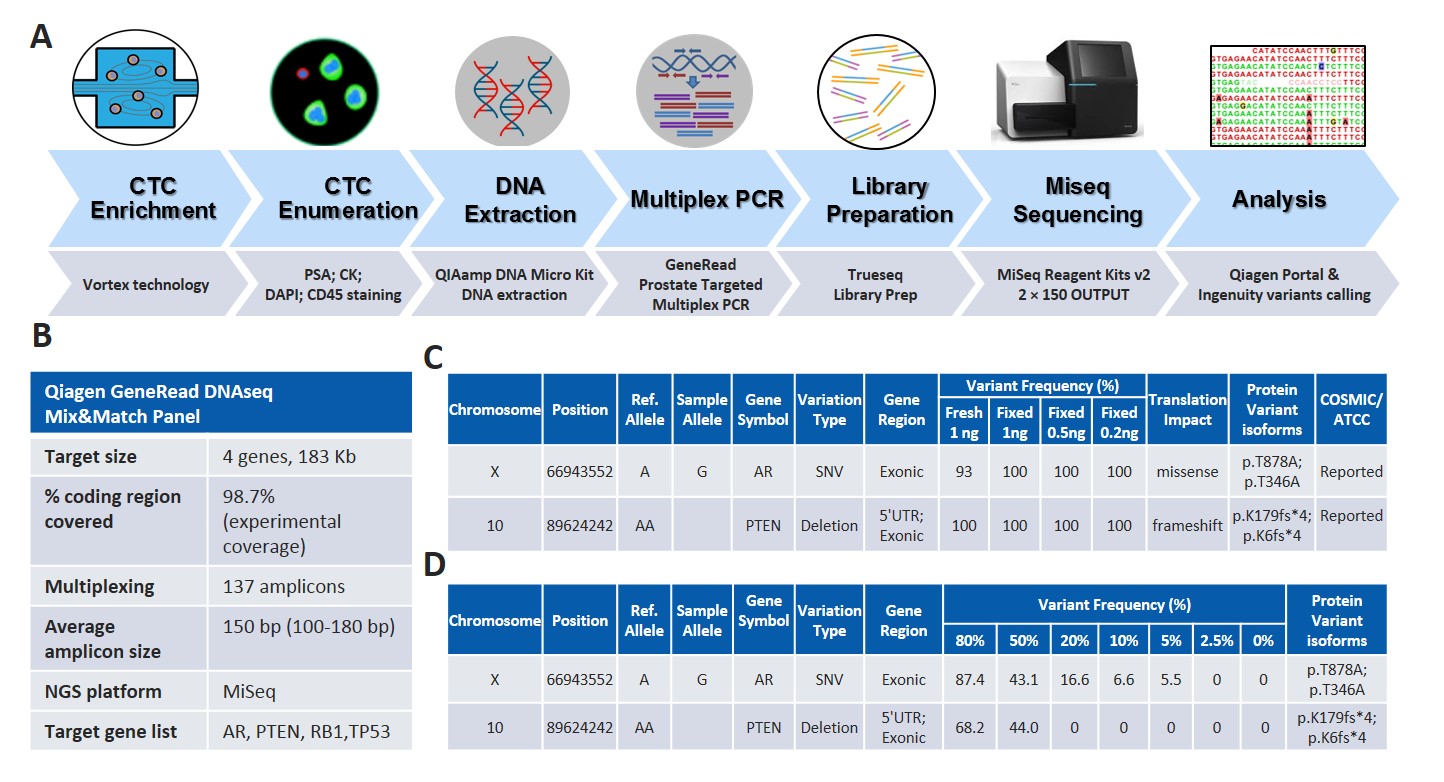

Supplement: Supplementary file 6 — Supplementary Figure 4 [file 41698_2017_15_MOESM6_ESM.jpg]
